# Objective and Automated Detection of Diffuse White Matter Abnormality in Preterm Infants Using Deep Convolutional Neural Networks

**DOI:** 10.3389/fnins.2019.00610

**Published:** 2019-06-18

**Authors:** Hailong Li, Nehal A. Parikh, Jinghua Wang, Stephanie Merhar, Ming Chen, Milan Parikh, Scott Holland, Lili He

**Affiliations:** ^1^The Perinatal Institute, Cincinnati Children’s Hospital Medical Center, Cincinnati, OH, United States; ^2^Department of Pediatrics, University of Cincinnati College of Medicine, Cincinnati, OH, United States; ^3^Department of Pediatrics, Nationwide Children’s Hospital, Columbus, OH, United States; ^4^Department of Radiology, University of Cincinnati College of Medicine, Cincinnati, OH, United States; ^5^Department of Electronic Engineering and Computing Systems, University of Cincinnati, Cincinnati, OH, United States; ^6^Medpace Inc., Cincinnati, OH, United States; ^7^Department of Physics, University of Cincinnati, Cincinnati, OH, United States

**Keywords:** diffuse white matter abnormality, very preterm infants, MRI, deep learning, convolutional neural networks

## Abstract

Diffuse white matter abnormality (DWMA), or diffuse excessive high signal intensity is observed in 50–80% of very preterm infants at term-equivalent age. It is subjectively defined as higher than normal signal intensity in periventricular and subcortical white matter in comparison to normal unmyelinated white matter on T_2_-weighted MRI images. Despite the well-documented presence of DWMA, it remains debatable whether DWMA represents pathological tissue injury or a transient developmental phenomenon. Manual tracing of DWMA exhibits poor reliability and reproducibility and unduly increases image processing time. Thus, objective and ideally automatic assessment is critical to accurately elucidate the biologic nature of DWMA. We propose a deep learning approach to automatically identify DWMA regions on T_2_-weighted MRI images. Specifically, we formulated DWMA detection as an image voxel classification task; that is, the voxels on T_2_-weighted images are treated as samples and exclusively assigned as DWMA or normal white matter voxel classes. To utilize the spatial information of individual voxels, small image patches centered on the given voxels are retrieved. A deep convolutional neural networks (CNN) model was developed to differentiate DWMA and normal voxels. We tested our deep CNN in multiple validation experiments. First, we examined DWMA detection accuracy of our CNN model using computer simulations. This was followed by *in vivo* assessments in a cohort of very preterm infants (*N* = 95) using cross-validation and holdout validation. Finally, we tested our approach on an *independent* preterm cohort (*N* = 28) to externally validate our model. Our deep CNN model achieved Dice similarity index values ranging from 0.85 to 0.99 for DWMA detection in the aforementioned validation experiments. Our proposed deep CNN model exhibited significantly better performance than other popular machine learning models. We present an objective and automated approach for accurately identifying DWMA that may facilitate the clinical diagnosis of DWMA in very preterm infants.

## Introduction

Diffuse white matter abnormality (DWMA) is observed in 50–80% of very preterm infants at term-equivalent age (Maalouf et al., 1999; Skiöld et al., 2010; Parikh et al., 2013). It is characterized by either (1) diffusely higher signal intensity in periventricular and subcortical white matter than in normal unmyelinated white matter on T_2_-weighted MRI images [also known as diffuse excessive high signal intensity (Skiöld et al., 2012; He and Parikh, 2013b)]; or (2) lower signal intensity than unmyelinated white matter on T_1_-weighted and fluid-attenuated inversion recovery (FLAIR) sequences. A number of prior studies (Maalouf et al., 1999; Counsell et al., 2003; Inder et al., 2003; Dyet et al., 2006; Krishnan et al., 2007; Cheong et al., 2009; Hagmann et al., 2009; Hart et al., 2010b; Skiöld et al., 2010; de Bruïne et al., 2011; Iwata et al., 2012; Jeon et al., 2012; He and Parikh, 2013a,b; Parikh et al., 2013) in the past two decades have reported the presence of DWMA in very preterm infants. Despite the well-documented presence of DWMA and emerging evidence of its pathological nature, the significance of DWMA for long-term neurodevelopment remains debatable (Dyet et al., 2006; Krishnan et al., 2007; Hart et al., 2010b; de Bruïne et al., 2011; Iwata et al., 2012; Jeon et al., 2012; He and Parikh, 2013a; Parikh et al., 2016; Volpe, 2017). Much of this debate has been fueled by the nearly universal use of qualitative reporting of DWMA that is subjective and unreliable, likely resulting in measurement error and lack of association with neurodevelopmental impairments in some studies (Hagmann et al., 2009; Hart et al., 2010a; de Bruïne et al., 2011). Volpe has speculated this finding to be a milder form of white matter injury that represents either periventricular leukomalacia with microscopic necrosis or isolated diffuse white matter gliosis (Volpe, 2017). The only DWMA imaging-pathologic correlation study reported some histopathologic overlap with periventricular leukomalacia, but also reported distinctive features, suggesting DWMA may be a form of diffuse white matter gliosis without microscopic necrosis (Parikh et al., 2016).

Only a few studies have attempted to develop reproducible quantitative methods for evaluating DWMA in preterm infants. Manually tracing DWMA regions on T_2_-weighted images, slice by slice, produces poor reliability and reproducibility (Hagmann et al., 2009; Hart et al., 2010a; de Bruïne et al., 2011). For example, the inter- and intra-observer agreement for visual diagnosis has ranged from a Kappa statistic of 0.14 to 0.44 (Hart et al., 2010a; Calloni et al., 2015), which is generally considered poor (Landis and Koch, 1977). The use of manual DWMA segmentation also significantly prolongs image processing time, limiting the utility of this approach for large studies (Yoshita et al., 2005). Accurate and automatic detection of DWMA is of crucial importance for resolving the debate about DWMA’s biologic nature and potentially risk stratifying high-risk preterm infants that may benefit from early intervention therapies (Hagmann et al., 2009; Mathur et al., 2010; Parikh, 2016). Limited studies have been published for automated detection of DWMA in infants (He and Parikh, 2013a,b; Parikh et al., 2013). These approaches were developed by utilizing only individual voxels for DWMA detection without considering the neighboring spatial information, which contributed to a higher false positive DWMA detection rate.

In adults, DWMA detection has been well investigated by using traditional machine learning techniques, including k-nearest neighbors (Griffanti et al., 2016), Bayesian models (Maillard et al., 2008), random forests (Geremia et al., 2011), logistic regression (Schmidt, 2017), and support vector machine (Lao et al., 2008). These machine learning approaches have been demonstrated to perform consistently well on T_1_-weighted or FLAIR MR images by taking advantage of spatial information of a given set of voxels (i.e., small image patches that are comprised of the given voxel and its neighboring voxels). These have enabled automated and objective detection of DWMA to facilitate epidemiological studies investigating the associations between DWMA and clinical outcomes (Guerrero et al., 2018). In recent years, studies using deep convolutional neural networks (CNN) and associated U-net architectures have outperformed traditional machine learning models in identifying DWMA in adults, due to CNN’s superior capacity in decoding complex image patterns (Brosch et al., 2013, 2016; Ghafoorian et al., 2016; Kamnitsas et al., 2017; Guerrero et al., 2018; Moeskops et al., 2018).

Deep CNN, inspired by the neuronal organization pattern of the visual cortex, is a class of deep learning models that have been widely applied in a range of machine learning tasks, such as image classification, natural language processing, and pattern recognition (LeCun and Bengio, 1995; LeCun et al., 1998, 2015). Compared to traditional approaches, CNN automatically extracts a hierarchy of increasingly complex image features from raw images without hand-engineered (i.e., unsupervised) feature extraction. This advantage is achieved by assembling a series of alternative operations as network layers into a consecutive multi-layer architecture. Although the individual layers only perform relatively simple operations such as convolution and pooling operations, the assembled CNN models are capable of mapping highly complex non-linearity between inputs and outputs. Various CNN architectures can be designed and modified for diverse machine learning tasks (Bengio and LeCun, 2007; Hinton et al., 2012; Krizhevsky et al., 2012; LeCun et al., 2015; Szegedy et al., 2015; Xu et al., 2015). Segmentation of DWMA on brain images could be implemented in two ways. A popular way is U-net based approaches, which take relatively large patches of original images. These have been applied on the adult applications with T_1_-weighted or FLAIR MR images. But, the performance of U-net approaches are still not desirable. Guerrero et al. (2018) reported a 69.5 of Dice score in their recent work. Another way is to apply CNN approaches on small images patches so as to classify individual voxels (Zhang et al., 2015). Considering the small number of sample size and low contrast on T_2_-weighted MR images in neonatal studies, we set to purse the second way in this work.

To fill the gap in accurate neonatal DWMA detection, we developed a deep learning approach to automatically identify DWMA regions on T_2_-weighted MRI images. Specifically, the detection of DWMA was formulated as an image voxel classification task. Small image patches that are centered on the given voxels were utilized to represent regional spatial information of individual voxels. A CNN model with the batch normalization technique was developed to differentiate normal white matter from DWMA voxels. The deep CNN architecture consists of feature extraction layers that aim to capture discriminative image patterns and high-level reasoning layers that are designed for decision making. We evaluated the proposed model using computer simulation, as well as internal and external validation using data from two independent very preterm infant cohorts.

## Materials and Methods

### Subjects

The data for this study was derived from two independent cohorts of very preterm infants. The Institutional Review Board of Nationwide Children’s Hospital (NCH) approved both studies and written parental informed consent was obtained for every subject. Infants with known structural congenital central nervous system anomalies, congenital chromosomal anomalies, congenital cyanotic cardiac defects, or overt brain injury were excluded. In addition, parents were not approached for consent if their infant remained on persistently high mechanical ventilator support (e.g., peak inspiratory pressure >30 and/or fraction of inspired oxygen >50%). All subjects were scanned with a brain MRI at term-equivalent age during natural sleep without the use of any sedation, after being fed and swaddled. MRI noise was minimized using Insta-Puffy Silicone Earplugs (E.A.R. Inc., Boulder, CO.) and Natus Mini Muffs (Natus Medical Inc., San Carlos, CA, United States).

#### Cohort 1

This cohort included 95 very preterm infants, ≤32 weeks gestational age that were recruited from four Columbus, Ohio area neonatal intensive care units, including NCH, Ohio State University Medical Center, Riverside Methodist Hospital, and St. Ann’s Hospital. We collected anatomical axial T_2_-weighted MRI images from each subject using the following sequence parameters: Repetition time (TR)/ echo time (TE) = 9,500/147 ms, flip angle (FA) = 90°, imaging matrix = 156 × 192, resolution 0.9 mm^3^× 0.9 mm^3^× 1.1 mm^3^ – on a 3T Siemens MAGNETOM Skyra scanner at NCH. Subjects from non-NCH sites had to be discharged from the NICU by term-equivalent age so they could be imaged at NCH. We used data from this cohort for deep CNN model development, internal cross-validation and holdout validation.

#### Cohort 2

This cohort included 28 very preterm infants, ≤32 weeks gestational age, all cared for in the neonatal intensive care unit at NCH (He et al., 2018). Anatomical scans were obtained with a proton density/T_2_-weighted sequence (TR/TE1/TE2 = 11,000/14/185 ms, FA = 90°, resolution 0.35 mm^3^ × 0.35 mm^3^× 2 mm^3^) on a 3T GE HDX scanner. We used data from this cohort for external validation.

Our inclusion criteria of very preterm infants born at 32 weeks gestational age or younger was selected based on the highest risk group for DWMA. The age range for our two cohorts was 23–32 weeks. Infants more mature than 32 weeks gestational age have a much lower incidence of DWMA and were therefore not included in the study/analyses. We selected a window of 39–44 weeks postmenstrual age for MRI scanning because this is the peak postmenstrual age when DWMA is observed on T_2_-weighted MRI (observed in 89% of very preterm infants between 40 and 44 weeks postmenstrual age in the cohort by de Bruïne et al. (2011). In this cohort, it was also found to be absent in infants imaged after 50 weeks postmenstrual age, thus confirming our choice of MRI timing. Demographics information for both cohorts is listed in Table 1.

**Table 1:** Baseline demographic information for both very preterm cohorts.

	Cohort 1	Cohort 2
Number of subjects	95	28
Sex	51M (53.7%)	14M (50%)
Birth weight (g)	1136.9 ± 397.5	979 ± 302.1
GA at birth (weeks)	28.5 ± 2.5	26.8 ± 2.1
PMA at scan (weeks)	40.4 ± 0.6	39.4 ± 1.3
Scanner	Siemens	GE

*GA, gestational age; PMA, post menstrual age; M, male; F, female. All ± data is mean ± SD.*

### Overview of DWMA Detection Using Deep CNN

**FIGURE 1:**
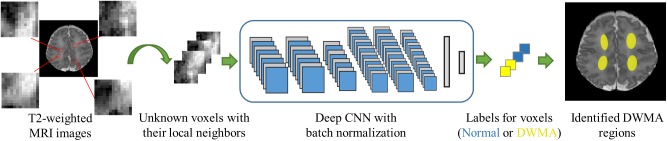
Overview of DWMA detection using deep CNN model.

We formulated the detection of DWMA into an image voxel classification task. Each T_2_-weighted white matter voxel is exclusively assigned into either DWMA or normal group. To utilize the image spatial information around voxels (Zhang et al., 2015), a small neighborhood/image patch centered on a given voxel is sampled. This typically results in a set of ∼10^5^ image patches for each subject. The deep CNN model takes each image patch as input and assigns a label to its center voxel (Figure 1).

### Deep CNN Architectures

**FIGURE 2:**
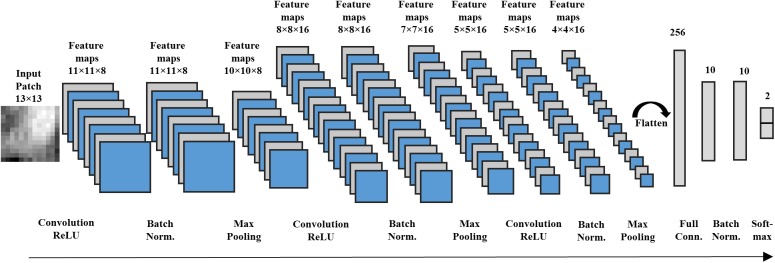
A deep CNN with 12 layers designed for image patches with size 13 × 13 voxels.

We designed a 12-layer deep CNN architecture, based on a prior study (Zhang et al., 2015), for image patches of 13 × 13 (Figure 2). The first hidden layer is a convolutional layer that contains 8 convolutional neurons. Each convolutional neuron consists of a trainable 2D filter of size 3 × 3 and a rectified linear unit activation function. Given an input **X** (i.e. a 2D n × n image patch), the activation output *a*_conv_ of the convolutional neuron can be represented by:

$$a_{\text{conv}}=\max(0,\;X*W_{\text{conv}})$$

where ∗ denotes the convolution operator and **W**_conv_ is the trainable weight map of the 2D filter. By using a stride size of one, the first hidden layer outputs eight 11 × 11 feature maps. The second hidden layer is a batch normalization layer, which performs a *batch normalizing transform* BN_γ,β_. Since a mini-batch stochastic gradient descent algorithm is applied to optimize the proposed CNN, this normalization step transforming on a mini-batch *B* = [*x*_1,…,m_] with *m* activation values can be described as:

$${\text{BN}}_{\gamma ,\beta }(x_{\text{i}})=\gamma \frac{x_{\text{i}}-\mu_{\text{B}}}{\sqrt{\sigma_{\text{B}}^{2}+\epsilon }}+\beta$$

where μ_B_ and σ_B_ are mini-batch mean and variance of mini-batch *B*. *x*_i_ is a particular activation of 2D feature maps. Parameters γ and β control the scale and shift of the normalized values, which are to be optimized during model training. ϵ is a small fuzz f number to avoid dividing by zero. Those 8 feature maps from the first hidden layer are normalized individually. The output of the second hidden layer are the normalized feature maps, which have the same size (e.g., 11 × 11) as the output of the previous layer. The third hidden layer is a max pooling layer, which combines the activation values of neuron clusters at prior layer into a single neuron by using the maximum value of the given cluster. We also applied a stride size of one for max pooling operation. This layer generates 8 feature maps with a size of 10 × 10. In the following, we applied convolutional, batch normalization and max pooling layers consecutively in this CNN architecture from the fourth to ninth hidden layers for feature extraction.

After obtaining sixteen 4 × 4 high-level feature maps, we flattened the feature maps into a single feature vector with 256 dimensions. Then, this feature vector is linked to the tenth hidden layer, a fully connected layer with 10 neurons. We also utilized a rectified linear unit activation function in the neurons of the fully connected layer. Assume that each neuron of the fully connected layer has a weight vector **W**_full_. Because the batch normalization layer is connected in the next layer, the bias **b** of neurons are removed. Given a flattened feature vector **v**, the activation of each neuron can be presented by:

$$a_{\text{full}}=\max(0,\;v\cdot W_{\text{full}})$$

where ⋅ indicates the dot product between vectors. The 10th hidden layer transforms the flattened feature vector with 256 dimensions into a new feature vector with 10 dimensions, functioning as a dimension reduction for the features. Again, a batch normalization layer, as the eleventh hidden layer, is applied to normalize the low-dimension feature vector. At the end, the normalized 10-dimension feature vector is input into a 2-way softmax layer (Bengio and LeCun, 2007) (i.e., the output layer) that produces the probability for the normal and DWMA groups. The proposed deep CNN for input patch 13 × 13 has 12 network layers, including a total of 6,264 trainable parameters.

Similarly, we designed different deep CNN architectures for other n × n image patches [n= 7,9,13,17] based on prior work (Zhang et al., 2015). Intuitively, larger input patches contain more neighboring spatial information, requiring a deeper network and more kernels for feature extraction. In contrast, smaller input patches need a shallower CNN and fewer convolutional kernels. The details of different architectures are listed in Table 2.

**Table 2:** Details of four deep CNNs for varying sizes of image patches.

Patch	Hyperparameters	Layer 1	Layer 2	Layer 3	Layer 4	Layer 5	Layer 6	Layer 7	Layer 8	Layer 9	Layer 10	Layer 11	Layer 12
7 × 7	Layer type	Conv.	Norm.	Pooling	Conv.	Norm.	Pooling	Full conn.	Norm.	Softmax	–	–	–
	Filter size	3	–	2 × 2	3	–	2 × 2	10	–	2	–	–	–
	Num of filter	8	–	–	8	–	–	1 × 1	–	–	–	–	–
9 × 9	Layer type	Conv.	Norm.	Pooling	Conv.	Norm.	Pooling	Full conn.	Norm.	Softmax	–	–	–
	Filter size	3	–	2 × 2	3	–	2 × 2	10	–	2	–	–	–
	Num of filter	8	–	–	16	–	–	1 × 1	–		–	–	–
13 × 13	Layer type	Conv.	Norm.	Pooling	Conv.	Norm.	Pooling	Conv.	Norm.	Pooling	Full conn.	Norm.	Softmax
	Filter size	3	–	2 × 2	3	–	2 × 2	3	–	2 × 2	10	–	2
	Num of filter	8	–	–	8	–	–	16	–	–	1 × 1	–	–
17 × 17	Layer type	Conv.	Norm.	Pooling	Conv.	Norm.	Pooling	Conv.	Norm.	Pooling	Full conn.	Norm.	Softmax
	Filter size	3	–	2 × 2	3	–	2 × 2	3	–	2 × 2	10	–	2
	Num of filter	8	–	–	16	–	–	16	–	–	1 × 1	–	–

*Conv, convolutional layer; Norm, batch normalization layer; Full conn, fully connected layer.*

### Deep CNN Training

We adopted cross-entropy as a loss function to train our deep CNN model. Assuming that *p* (*y*_i_|**X**_i_; **W**) and *y*_i_ are the predicted and true probability values for *i*^th^ image patch **X**_i_, the loss function for *N* training samples is calculated by:

$$\begin{array}{c} H(W)=-\frac{1}{N}\sum_{\text{i}=1}^{\text{N}}y_{\text{i}}\log(p(y_{{}_{\text{i}}}|X_{\text{i}};\;W))\\ \;+(1-y_{\text{i}})\log(1-p(y_{\text{i}}|X_{\text{i}};\text{ W})) \end{array}$$

A mini-batch stochastic gradient descent algorithm (Johnson and Zhang, 2013) was chosen to minimize the above loss function so as to optimize the weights **W** of deep CNN. This algorithm divides the training data into small batches and updates the network weights using only data from every batch. It enables a faster, but more stable convergence for model training. We configured batch size as 256. To further accelerate the training, we applied a Nesterov momentum technique (Nesterov, 2007) for parameter searching. The weights of convolutional and fully connected layers were randomly initialized using Glorot uniform distribution (Glorot and Bengio, 2010). The learning rate was set as 0.1 based on classification performance after testing several empirical values [0.001, 0.01, 0.1, 0.5]. The number of epochs was set as 20 with an early stop mechanism, which would cease the optimization process if three consecutive epochs return the same loss errors.

### Model Evaluation

DWMA gold standard information was annotated by two experts guided by an atlas-based method (He and Parikh, 2013a). All T2-weighted MRI data were obtained in Digital Imaging and Communications in Medicine (DICOM) format from two IRB-approved prospective studies (Table 1). We transferred MRI data into the Neuroimaging informatics technology Initiative (NIfTI) format. Typical procedure of Anterior Commissure (AC)-Posterior Commissure (PC) correction for each subject was performed using Statistical Parametric Mapping (SPM) package (Friston, 1994). We further conducted skull-stripping and tissue segmentation by using a neonatal structural MRI processing pipeline (He et al., 2018). Tissue probability maps for white matter, gray matter and cerebrospinal fluid voxels of T_2_-weighted images were obtained. We normalized T_2_-weighted images by using the z-score transformation. After preprocessing, DWMA regions of T_2_-weighted images were outlined by identifying the white matter voxels with greater than or equal to α = 1.4 standard deviation (SD) above the mean for cerebral tissues. All DWMA false positive voxels in the detected regions and isolated false positive voxels were manually corrected. Two DWMA expert raters evaluated the images individually, then collaborated to conclude a gold-standard DWMA dataset. Compared to normal voxels, the number of DWMA voxels are relatively small, therefore this results in an imbalanced classification problem (a disproportionate ratio of observations in each class). We therefore applied Dice index (Dice, 1945) and balanced accuracy (Brodersen et al., 2010) for the model evaluation on individual testing subjects. Given two sets, A and B, the Dice index is defined as:

$$\mathrm{Dice}(A,\;B)=\frac{2|A\cap B|}{\left|A|+|B\right|}$$

where |∗| denotes the number of elements in a set. The Dice index is a real number in [0, 1], where a larger value indicates a higher similarity between automatically detected DWMA regions and gold standard regions. We denote true positive as *TP*, representing the number of correctly classified samples among positive samples *P*; and true negative as *TN*, representing the number of correctly classified samples among *N* negative samples. Then, balanced accuracy is defined by:

$$\mathrm{Balanced Accuracy}=\frac{1}{2}\left(\frac{\text{TP}}{P}+\frac{\text{TN}}{N}\right)$$

Balanced accuracy measures the average accuracy obtained from both the minority and majority classes. It is equivalent to the traditional accuracy if a model performs equally well on either classes. Conversely, it avoids “falsely” high value due to the model taking advantage of the distribution of the majority class.

To compare the proposed deep CNN model with other popular machine learning models, we developed deep neural network (DNN) and support vector machine (SVM) models. The DNN architecture design is displayed in the Supplementary Table. An SVM classifier was implemented, as suggested in Zhang et al. (2015), using a linear kernel for neonatal brain image segmentation. To optimize the SVM model, the soft margin *C* was selected via a linear search from a set of empirical values [i.e., *C* = (2^−10^, 2^−8^, ..., 1, ....2^8^, 2^10^)]. The soft margin was determined optimal when the DWMA detection performance of the model on the testing data was maximal. The DNN and SVM models were configured and optimized with flattened vectors of image patch sizes, individually. *T*-test was applied to test whether there is a significant difference (*p* < 0.05) between the mean performances of two models.

## Results

### Computer Simulation

**FIGURE 3:**
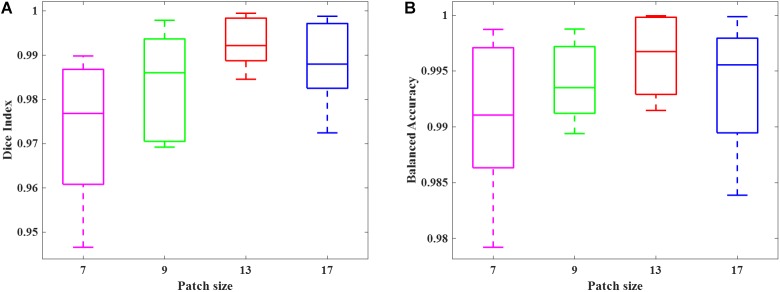
Box plots of the DWMA detection performance, including **(A)** Dice index and **(B)** balanced accuracy using four different deep CNN architectures tested on 10 simulated preterm neonatal brain images. The central line indicates the median, and edges of the box indicate the 25 and 75th percentiles. The whiskers extend to the maximum and minimum values.

We simulated 10 neonatal T_2_-weighted brain images with manually drawn synthetic DWMA regions using a method presented in our previous study (He and Parikh, 2013b). Rician noise (SD = 10) was imposed on the simulated images. The signal-to-noise ratio (SNR), defined as the mean cerebral tissue intensity divided by noise SD, of the synthesized brain images was 22.5. Four deep CNN models (Table 2) were implemented to detect these synthetic DWMA regions. We applied a leave-one-subject-out cross-validation strategy to evaluate the models. The detection performance of four CNN architectures are displayed in the Figure 3 box plots. We observed that deeper architectures for the larger patch sizes were generally better than ones for smaller sizes and the CNN architecture for patch size 13 × 13 was slightly more accurate than for patch size 17 × 17.

**FIGURE 4:**
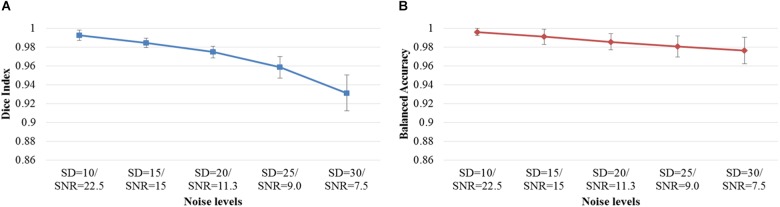
DWMA detection performance, including **(A)** Dice index and **(B)** balanced accuracy of the 12-layer deep CNN model with input patch size of 13 × 13 on 10 simulated preterm neonatal brain images with varying noise levels. The error bars indicate the SD of performance. Increasing noise levels only marginally affect DWMA detection performance. Noise levels were displayed with simulated Rician noise standard deviation (SD) and image signal-to-noise ratio (SNR).

**FIGURE 5:**
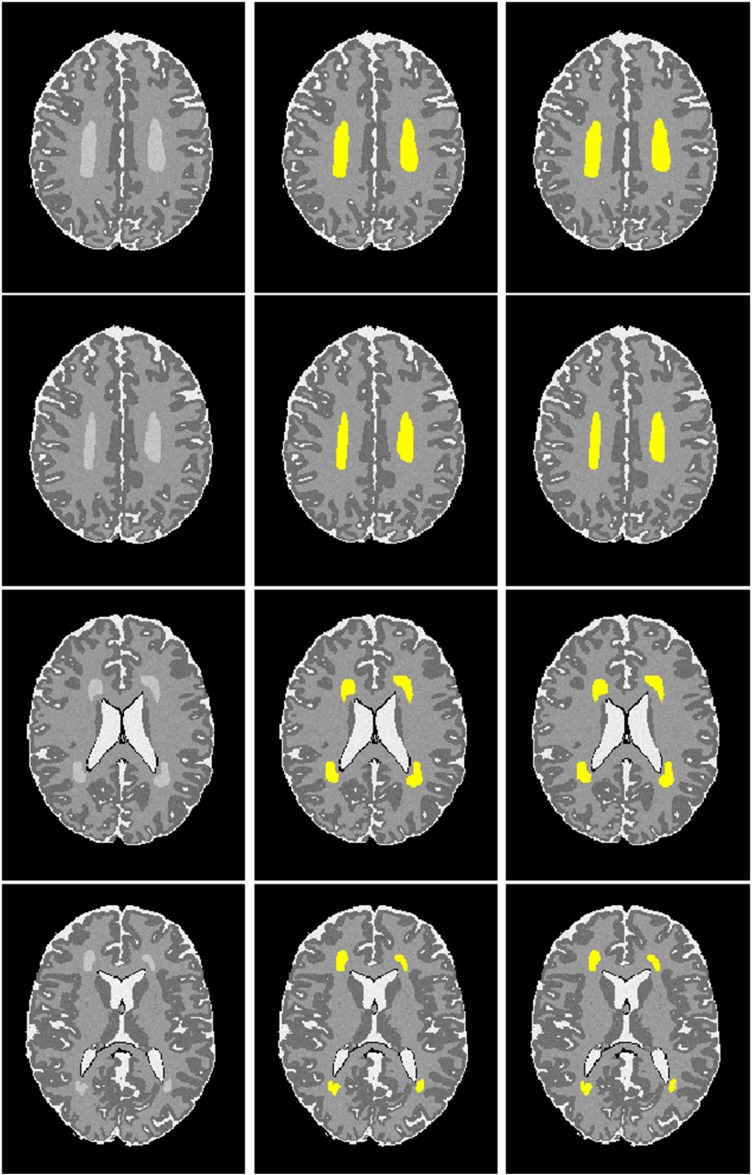
Visualization of automated DWMA detection on simulated preterm neonatal MRI images using a 12-layer deep CNN. Left column, simulated images in axial orientation at the level of the centrum semiovale and lateral ventricles; Middle column, images with outlined ground truth (synthetic DWMA); Right column, images with CNN-detected DWMA.

Next, we examined the Dice index and balanced accuracy of the 12-layer deep CNN model for patch size 13 × 13 across 10 subjects with varying noise levels (Figure 4). Different Rician noise [*SD* = (10, 15, 20, 25, and 30)] was added into the synthetic images, whose corresponding SNR were [22.5, 15, 11.3, 9.0, and 7.6], respectively. Deep CNN was able to achieve the Dice index (mean ± SD, 0.993 ± 0.006) and balanced accuracy (0.996 ± 0.004) when SNR = 22.5. As noise levels were increased, the detection performance of deep CNN decreased, but only marginally. When SNR = 7.6, the deep CNN model achieved 0.931 ± 0.019 for Dice index and 0.976 ± 0.014 for balanced accuracy Figure 5 shows that the deep CNN-identified brain regions strongly overlap with ground truth.

### *In vivo* Data

#### Internal Cross-Validation

**FIGURE 6:**
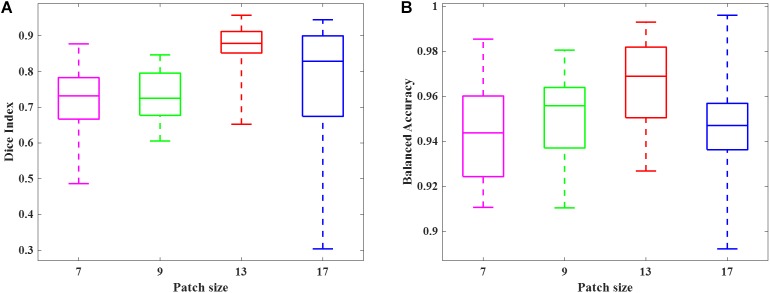
Box plots of DWMA detection performance, including **(A)** Dice index and **(B)** balanced accuracy, using four different deep CNN architectures on 50 very preterm neonatal brain images. The central line indicates the median, and edges of the box indicate the 25 and 75th percentiles. The whiskers extend to the maximum and minimum values.

We randomly selected 50 subjects from cohort 1 and conducted a 10-fold cross-validation scheme to validate the deep CNN model using preterm infants’ MRI data. The 50 subjects were randomly divided into 10 equal sized portions. For each iteration, 5 subjects (∼5 × 10^5^ image patches) were held out for the model testing, and the remaining 45 subjects (∼45 × 10^5^ image patches) were used for model training. This process was repeated for 10 iterations until each of the 10 portions was evaluated once as the testing data. We first compared the DWMA detection performance of four deep CNN architectures (Table 2) and reported the Dice index and balanced accuracy for each subject using box plots (Figure 6). As we found for the computer simulation (Figure 3), the 12-layer CNN designed for patch size 13 × 13 achieved more accurate detection performance than other architectures.

**FIGURE 7:**
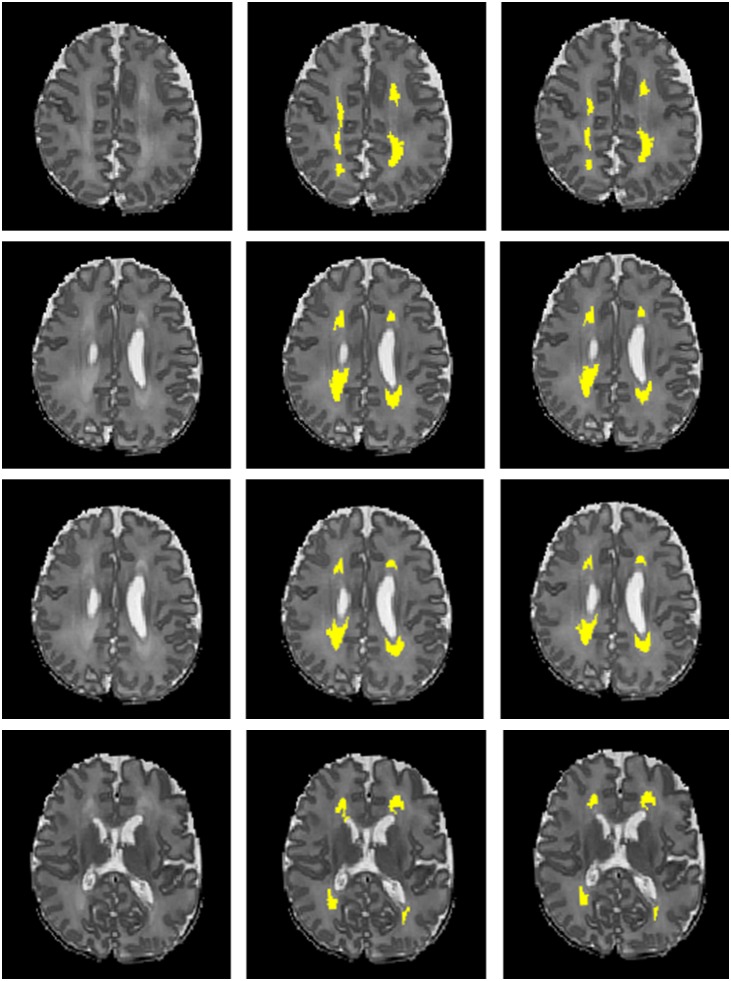
A male subject born at age 28.3 weeks and imaged at term-equivalent age exhibiting DWMA (highlighted in yellow) in periventricular white matter regions on T_2_-weighted brain images. Left column, 4 axial levels of T_2_-weighted images; Middle column, segmented images with gold standard DWMA; Right column, images with deep CNN-detected DWMA.

Next, we compared the proposed 12-layer deep CNN with DNN and SVM models. Table 3 shows the DWMA detection performance using these different models. The CNN model exhibited a significantly higher Dice index than DNN (*p* = 0.019) and SVM (*p* < 0.001). The balanced accuracy for CNN was also significantly higher than that of DNN (*p* = 0.043) and SVM (*p* < 0.001). Figure 7 displayed a representative DWMA detection using deep CNN. The automatically detected DWMA closely approximated the DWMA gold standard regions confirmed by human experts (both highlighted in yellow).

**Table 3:** Cross-validation.

	Dice	Balanced accuracy
CNN	**0.864 (0.052)**	**0.942 (0.028)**
DNN	0.831 (0.122)	0.922 (0.018)
SVM	0.818 (0.115)	0.895 (0.071)

*Mean (SD) Dice index and balanced accuracy of the three machine learning models for DWMA detection. The proposed CNN model outperformed compared DNN and SVM in the 10-fold cross-validation with 50 subjects. CNN, convolutional neural networks; DNN, deep neural networks; SVM, support vector machine. The boldface denotes the best performance for individual metrics in the validation experiment.*

**FIGURE 8:**
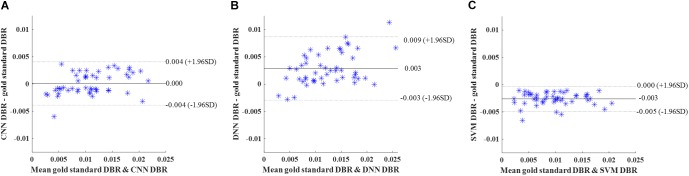
Cross validation. Bland-Altman plots of gold standard DWMA-to-brain-ratio (DBR) and automatic DBRs: **(A)** convolutional neural networks (CNN), **(B)** deep neural networks (DNN), and **(C)** support vector machine (SVM) in the 10-fold cross-validation with 50 subjects. Each blue asterisk represents one subject. Solid horizontal lines represent the mean difference and dashed lines represent the limits of agreement (±1.96 SD).

Then, we calculated DWMA volumes – a prognostic biomarker that has been shown to be a significant predictor of later cognitive scores (Dyet et al., 2006; Krishnan et al., 2007; Iwata et al., 2012; He and Parikh, 2013a) – based on detection using the deep CNN, DNN, and SVM. DWMA volumes were normalized by head size, denoted as DWMA-to-brain-ratio [DBR = DWMA volume divided by total brain volume (He and Parikh, 2013a)]. Bland-Altman plots were utilized to assess the degree of agreement between the automatic and gold standard DBRs (Figure 8). The mean difference between CNN and gold standard DBRs was near zero (1.007E-04). Compared to this, mean difference between gold standard and the other two automatic DBRs were more than one order of magnitude larger (DNN: +0.003 and SVM: −0.003).

#### Internal Holdout Validation

We further compared our CNN with DNN and SVM models using internal holdout validation. We trained models using 50 randomly selected subjects (∼50 × 10^5^ image patches) in cohort 1 (*N* = 95) and tested the models on the remaining 45 subjects (∼45 × 10^5^ image patches) from the same cohort. Table 4 highlights the higher performance of the CNN model over the DNN and SVM models with a mean Dice index of 0.859 and a mean balanced accuracy of 0.924. CNN exhibited a significantly higher Dice ratio than DNN (*p* = 0.027) and SVM (*p* = 0.036). In addition, balanced accuracy of CNN was also higher than for DNN (*p* < 0.001) and SVM (p < 0.001) models.

**Table 4:** Holdout validation.

	Dice	Balanced accuracy
CNN	**0.859 (0.098)**	**0.924 (0.06)**
DNN	0.817 (0.109)	0.905 (0.033)
SVM	0.806 (0.093)	0.885 (0.037)

*Mean (SD) Dice index and balanced accuracy of the three machine learning models for DWMA detection. The proposed CNN model achieved better performance than DNN and SVM in the internal holdout validation with 50 subjects as training set and 45 subjects as holdout testing set. CNN, convolutional neural networks; DNN, deep neural networks; SVM, support vector machine. The boldface denotes the best performance for individual metrics in the validation experiment.*

**FIGURE 9:**
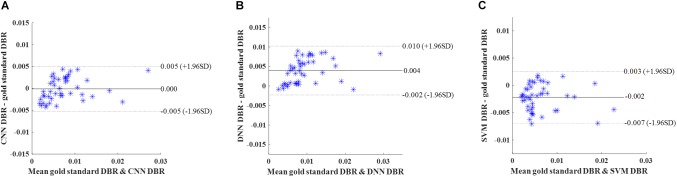
Internal holdout validation. Bland-Altman plots of gold standard DWMA-to-brain-ratio (DBR) and automatic DBRs: using **(A)** convolutional neural networks (CNN), **(B)** deep neural networks (DNN), and **(C)** support vector machine (SVM) in the internal holdout validation with 50 subjects as training set and 45 unseen subjects as holdout testing set. Each blue asterisk represents one subject. Solid horizontal lines represent the mean difference and dashed lines represent the limits of agreement (±1.96 SD).

Similar to cross-validation, we calculated DBR of each subject based on detection using the deep CNN, DNN, and SVM. Bland-Altman plots were utilized to assess the degree of agreement (Figure 9). The mean difference between CNN and gold standard DBRs was near zero (1.217E-04). The DBR of our CNN outperformed other two automatic DBRs (DNN: +0.004 and SVM: −0.002).

#### External Independent Validation

Last, in order to evaluate the robustness and generalizability of our methods, we tested our models on an independent dataset that was obtained using a different MRI scanner. The models trained using 50 subjects (∼50 × 10^5^ image patches) from cohort 1 were tested on this independent cohort 2 with 28 subjects (∼28 × 10^5^ image patches). The CNN performance remained robust and once again significantly outperformed DNN (*p* = 0.018 for Dice ratio; *p* = 0.009 for balanced accuracy) and SVM (*p* = 0.021 for Dice ratio; *p* = 0.006 for balanced accuracy) (Table 5).

**Table 5:** External validation.

	Dice	Balanced Accuracy
CNN	**0.845 (0.079)**	**0.874 (0.065)**
DNN	0.788 (0.075)	0.836 (0.067)
SVM	0.786 (0.077)	0.832 (0.056)

*Mean (SD) Dice index and balanced accuracy of the three machine learning models for DWMA detection. The proposed CNN model outperformed compared DNN and SVM in the external validation with 50 subjects from Siemens scanner as training set and 28 subjects from GE scanner as independent testing set. CNN, convolutional neural networks; DNN, deep neural networks; SVM, support vector machine. The boldface denotes the best performance for individual metrics in the validation experiment.*

**FIGURE 10:**
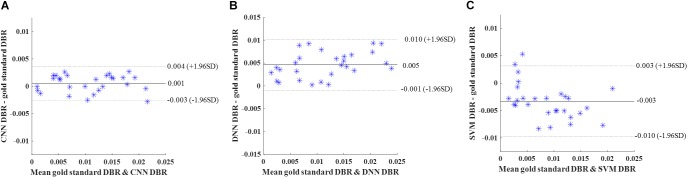
External validation. Bland-Altman plots of gold standard DWMA-to-brain-ratio (DBR) and automatic DBRs: **(A)** convolutional neural networks (CNN), **(B)** deep neural networks (DNN), and **(C)** support vector machine (SVM) in the external validation with 50 subjects as training set and 28 independent subjects as testing set. Each blue asterisk represents one subject. Solid horizontal lines represent the mean difference and dashed lines represent the limits of agreement (±1.96 SD).

Again, DBR based on detection using the deep CNN, DNN, and SVM were calculated for individual subjects. Bland-Altman plots were used (Figure 10). The mean difference between CNN and gold standard DBRs (0.001) was smaller than the ones between two compared automatic DBRs and god standard DBRs (DNN: +0.005 and SVM: −0.003).

## Discussion

We present a deep CNN approach to objectively and automatically quantify DWMA regions on T_2_-weighted MRI images. This is the first study to detect DWMA regions and quantify associated volumes in preterm infants by using a state-of-the-art deep learning algorithm. The excellent image pattern recognition capability of deep CNN enabled our proposed approach in automated detection of DWMA with a detection level similar to human experts. The desirable generalizability of our approach, tested on two preterm cohorts and scanner platforms, suggests that we can achieve consistent and reliable diagnosis of DWMA.

To date, the diagnosis of DWMA in preterm neonates has lacked sufficient reliability, even by trained neuroradiologists. Reported inter- and intra-observer agreement for qualitative diagnosis of DWMA is poor (Hart et al., 2010a). Technical variations such as imaging protocols and platforms may contribute to the difficulty of consistent DWMA detection. Moreover, DWMA diagnosis can also be confounded by the developmental crossroad regions in frontal and occipital periventricular white matter that contain multiple crossing fibers and rich extracellular matrix (Judaš et al., 2005; Kidokoro et al., 2011). These confounding factors could partly explain the conflicting reports of significant association (Dyet et al., 2006; Krishnan et al., 2007; Iwata et al., 2012; He and Parikh, 2013a) vs. no association with cognitive outcomes (Jeon et al., 2012), and hypotheses about whether DWMA represents a developmental delay or pathologic lesions (Counsell et al., 2003; Inder et al., 2003; Krishnan et al., 2007; Cheong et al., 2009; Hagmann et al., 2009; Hart et al., 2010b; He and Parikh, 2013a). Our experiments on computer simulated and *in vivo* data suggest that the proposed deep CNN approach can yield reproducible DWMA diagnosis across different cohorts.

The computer simulation experiments provided theoretical support to the validity of the proposed approach. Unlike gold standard data that is derived by human experts, the ground truth of DWMA regions in computer-simulated brain images is well grounded. Additionally, confounding factors such as imaging protocol, scanner configuration, and subject motion are not a concern when using computer simulated images. Our proposed CNN approach detected DWMA regions with high accuracy and its tolerance to varying signal noise levels was high. Although noise level for a clinical neonatal MRI scan is dependent on scanner and environment, a generally acceptable noise SD is less than 25 (He and Parikh, 2013b). At such a noise level (α = 25), the CNN model still achieved a very high Dice index (0.96) and balanced accuracy (0.98).

The comparison of deep CNN architectures, utilizing the computer simulation and cross-validation experiments, demonstrated that deeper architectures perform better for DWMA detection. This is consistent with previous works (Bengio, 2009; Zhang et al., 2015; Goodfellow et al., 2016) on the general trend that a deeper architecture tends to perform better for complex image pattern recognition. Meanwhile, the CNN architecture for patch size 13 × 13 performed slightly better than the one for patch size 17 × 17, suggesting that simply increasing the size of patches may not further improve detection. Peak performance for detection of DWMA in infant brain images was achieved by the deep CNN with the patch size 13 × 13, which may be related to the spatial scale of the regional anatomy. Prior research (Kamnitsas et al., 2017) suggests that increasing the spatial scale may negatively impact the detection of the regional spatial patterns.

With respect to comparing machine learning models, the strong performance of deep CNN for image pattern recognition shown here is consistent with numerous prior image classification studies (Krizhevsky et al., 2012; de Brebisson and Montana, 2015; LeCun et al., 2015). Given sufficiently large training data, deep learning methods have outperformed traditional classifiers (e.g., SVM) in decoding complex image patterns (LeCun et al., 2015). As a specialized neural network, CNN further leverages the performance of DNN by using a convolution function, which improves the utilization of spatial information within images (Goodfellow et al., 2016).

Our experiments of internal holdout validation and external independent validation support the strong generalizability of our CNN approach. In the holdout validation, the performance of CNN on 45 holdout subjects from cohort 1 was comparable to the one achieved for cross-validation experiments. Compared to internal holdout validation, the external validation results for cohort 2 provided additional evidence that this method is generalizable to very preterm infants imaged on different scanners and using different imaging parameters.

Our current work has certain limitations. First, the performance of machine learning methods for automated detection and classification is highly dependent on the training data. Although we had a substantial data set available from very preterm infants, training the CNN with more data that has been classified by experts can be expected to improve performance. Second, although computer simulation and two independent cohorts from separate studies were utilized to evaluate our method, the i*n vivo* data were collected by the same research group (despite enrollment at different centers) and all images were acquired at a field strength of 3 Tesla, limiting the variability of tested MRI images. The sizes of our *in vivo* cohorts are relatively small. Additional data from other institutions or research groups, and possibly at other field strengths would provide further validation of the generalizability of our approach. Third, to ensure deep learning models had adequate training data, we utilized image patches, typically ∼10^5^ for each subject, as the training samples. However, this strategy introduced redundancy among overlapping patches, causing expensive computing cost. Fourth, although we strived to obtain a robust gold-standard dataset, it is worth mentioning that the inter-rater variability may be a source of bias in the evaluation of the proposed and peer models. Finally, our current CNN approach was developed based on T_2_-weighted images only. Additional imaging modalities (e.g., T_1_-weighted images) may further improve the accuracy of DWMA detection.

In summary, we developed a deep CNN approach for automated and objective DWMA detection. The experiments were conducted by applying the proposed method to T_2_-weighted anatomical images at term-equivalent age from very preterm infants. The computer simulations and internal and external validation demonstrated very accurate and reproducible DWMA detection performance that may facilitate the clinical diagnosis of DWMA in very preterm infants. Future studies to investigate the association between CNN-detected DWMA volumes and long-term neurodevelopmental outcomes, as we are currently doing, will be important to further validate the clinical significance of this work.

## Data Availability

The datasets for this manuscript are not publicly available because the IRB of national children’s hospital does not allow publication of preterm infant brain images. Requests to access the data sets should be directed to LH, lili.he@cchmc.org.

## Ethics Statement

This study was carried out in accordance with the recommendations of the Institutional Review Board of Nationwide Children’s Hospital (NCH) with written informed consent from all subjects. All subjects gave written informed consent in accordance with the Declaration of Helsinki. The protocol was approved by the Institutional Review Board of Nationwide Children’s Hospital.

## Author Contributions

HL, NP, and LH designed the study. HL, JW, MC, MP, and LH conducted the data processing and analysis. All authors wrote the manuscript.

## Conflict of Interest Statement

SH was employed by company Medpace. The remaining authors declare that the research was conducted in the absence of any commercial or financial relationships that could be construed as a potential conflict of interest.
