# Supplementary material for: Objective and Automated Detection of Diffuse White Matter Abnormality in Preterm Infants Using Deep Convolutional Neural Networks
Source: Front Neurosci. 2019 Jun 18;13:610. doi: 10.3389/fnins.2019.00610 (PMC6591530; doi:10.3389/fnins.2019.00610)
Supplement: Supplementary file 1 [file Table_1.DOCX]

**Supplemental Table.** Architecture of DNN models for varying patch sizes.

| Patch | Hyperparameters | layer 1 | layer 2 | layer 3 | layer 4 | layer 5 | layer 6 | layer 7 |
| --- | --- | --- | --- | --- | --- | --- | --- | --- |
| 7x7 | layer type | Full conn. | Norm. | Full conn. | Norm. | Softmax |  |  |
|  | Num of neuron | 30 | - | 10 | - | 2 |  |  |
| 9x9 | layer type | Full conn. | Norm. | Full conn. | Norm. | Softmax |  |  |
|  | Num of neuron | 50 | - | 10 | - | 2 |  |  |
| 13x13 | layer type | Full conn. | Norm. | Full conn. | Norm. | Full conn. | Norm. | Softmax |
|  | Num of neuron | 50 | - | 30 | - | 10 | - | 2 |
| 17x17 | layer type | Full conn. | Norm. | Full conn. | Norm. | Full conn. | Norm. | Softmax |
|  | Num of neuron | 50 | - | 30 | - | 10 | - | 2 |

Norm: batch normalization layer; Full conn: fully-connected layer.
